# Analytical modeling of gas production rate in tight channel sand formation and optimization of artificial fracture

**DOI:** 10.1186/s40064-016-2176-7

**Published:** 2016-04-27

**Authors:** Ruifei Wang, Hongqing Song, Hewei Tang, Yuhe Wang, John Killough, Gang Huang

**Affiliations:** College of Petroleum Engineering, Xi’an Shiyou University, Xi’an, Shaanxi China; Department of Petroleum, Texas A&M University, College Station, TX USA; Department of Petroleum, Texas A&M University at Qatar, Doha, Qatar; School of Civil and Environmental Engineering, University of Science and Technology, Beijing, China

**Keywords:** Tight sandstone gas, Channel sand formation, Permeability anisotropy, Analytical solution

## Abstract

Permeability variation in tight channel sand formation makes an important role in gas production. Based on the features of channel sand formation, a mathematical model has been established considering anisotropy of permeability. The analytical solutions were derived for productivity of both vertical wells and vertically fractured wells. Simulation results show that, gas production rate of anisotropic channel sand formation is less than that of isotropic formation. For vertically fractured well, artificial fracture direction, drainage radius, permeability ratio and fracture half-length have considerable influence on production rate. The optimum fracture direction should be deviated less than π/8 from the maximum permeability direction (or the channel direction). In addition, the analytical model was verified by in situ measured data. The research provides theoretical basis for the development of tight channel sand gas reservoirs.

## Background

With development of fracturing technology, unconventional oil and gas production are growing fast (Clarkson et al. 2012; Ahn et al. 2014; Shanley et al. 2004; Dai et al. 2012; Rivard et al. 2014). Tight gas reservoirs are widespread in many major basins in China such as the Ordos Basin, the Sichuan Basin, the Bohai Bay Basin, the Songliao Basin, and the Junggar Basin (Zhu et al. 2012). Tight sandstone gas reservoirs are characterized by low porosity, high heterogeneity, extensive hydrocarbon generation, short-distance petroleum migration and complex formation pressure system (Wu et al. 2012; Zou et al. 2014; Deveugle et al. 2014; Wei et al. 2014).

The typical tight sandstone gas reservoir, represented by braided river sedimentary sediments, has various sedimentary micro-facies including braided bar, channel filling, flood basin and crevasse splay. Effective reservoir is mainly coarse sand deposited in braided bar or channel filling. According to the core analysis data, those coarse sand in braided bar and channel filling has the excellent permeability (0.8–2.0 × 10^−3^ μm^2^) with 35–40 % thickness of the total sand. The sediment with braided bar micro-facies contributes about 75 % of the coarse sand and the rest are mostly from channel filling (Xie et al. 2015; Wei et al. 2014; Zhao et al. 2010). The sands deposited along the channel direction, which is the paleo-current direction, and have relatively high permeability (0.5–2.0 × 10^−3^ μm^2^). Sands deposited perpendicular to the direction of the channel has low permeability (0.06–1.0 × 10^−3^ μm^2^). This phenomenon demonstrates high permeability anisotropy in sand reservoir with fluvial deposition.

The permeability anisotropy is the basic property of channel sand formation, especially for those with coarse fluvial sand. It leads to certain negative influence on the reservoir development index prediction. Conventionally numerical simulation methods were implemented to model the impact of permeability anisotropy (Bai et al. 1993; Liu et al. 2013; Aghighi and Rahman 2010). The numerical simulation methods fall into two categories: (1) Numerical values of the three-dimensional three-phase anisotropy permeability are applied in simulation model. The finite difference method and complete implicit iterated method are used. (2) Stochastic simulation method based on geological statistics, which describes the anisotropy of permeability and obtains the effective permeability (Wang et al. 2011; Khan and Teufel 2000; Farrell et al. 2014; Fauzi 2011; Hajizadeh et al. 2011). There are numerous challenges to apply the numerical simulation in real cases. The simulation results are highly dependent on the mesh generation, while a coarse grid model brings uncertainty to detect and describe the geological heterogeneity (Song et al. 2015). In addition, in order to obtain an accurate prediction, it requires to adjust various factors for historical fitting. The high demand for calculation and adjusting parameters limits the further development of the numerical simulation method.

Analytical method has the advantages over numerical simulation method by directly deriving the relationship among major parameters. It simplifies the calculation procedure, with advanced methodology to describe the percolation theory and exploitation law (Zhang et al. 2008; Song et al. 2014). Currently, most analytical methods are derived in ideal conditions, which cannot reflect the influence of anisotropy of permeability.

What this paper intends to do are as follows: (1) based on the features of channel sand formation, establish a mathematical model considering artificial fracture; (2) derive analytical solutions for productivity of both vertical wells and vertical fractured wells; (3) analyze the influence of production parameters on production rate.

## Geological description and physical model

**Fig. 1:**
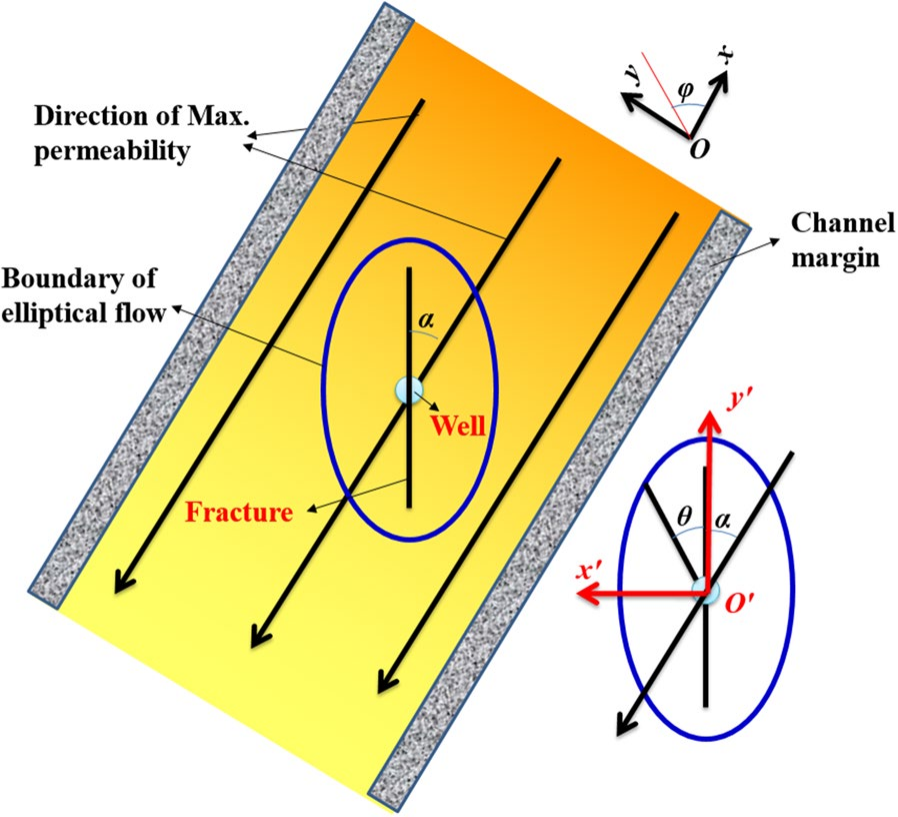
Physical model of channel sand formation

As known, sandstone is a type of sedimentary rock. Therefore, the permeability in the vertical direction is almost same if the thickness is small. The two-dimensional model was built in this paper. Figure 1 is a horizontal sketch map of the channel sand formation and shows the physical model. The permeability of the channel sand reservoir will be anisotropic due to the river flow direction. Commonly, the direction paralleling to the river flow will hold the largest permeability. On the contrary, the direction perpendicular to the river flow will hold the smallest permeability. We can get the maximum and minimum permeability of sandstone. Then the permeability distribution at various directions also can be calculated.

If the vertical well was used to exploit gas from the sandstone gas reservoir, it is easy to use Darcy’s Law to calculate the production rate. However, gas flow in fracture may not satisfy the Darcy’s Law. As shown in Fig. 1, the elliptical flow could play an important role in the performance of fractured tight gas well. We use elliptical flow to reflect the non-Darcy effect in artificial fracture. If fracturing is applied to enhance the gas recovery, the final production rate will be influenced by the direction of fracture (α) due to the anisotropy of the permeability. Fracture direction α is the angle between fracture and the direction of largest permeability in the x–y coordinate system.

In order to get the analytical solutions of differential equations modeling flow in vertical fractured well, two coordinate systems were set up. The x–y coordinate system is the absolute coordinate system, and the x-axis is paralleling to the direction of largest permeability. The other system x′–y′ is the ellipse coordinate, and the x-axis is paralleling to the long axis of ellipse. In addition, $\varphi$ is the angle between fracture and x-axis in the x–y coordinate system, and θ is the angel fracture and y′-axis in the x′–y′ coordinate system. Obviously, $\varphi$ is the summation of θ and α.

To investigate the influence of permeability characteristics of channel sand formation on the production rate, we will consider the following two cases: one is vertical well without fractures, and the other is vertical fractured well. Basic assumptions are made to derive the mathematical models:

1. The reservoir is anisotropic and horizontally infinite with uniform thickness of *h*.
2. The gas flows in terms of planar flow in steady state with constant viscosity.
3. The fracture has negligible width and uniform height the same to reservoir thickness.

## Mathematical model and analytical solutions

### Production rate of a vertical well

According to mass conservation, for planar radial flow

1 $$\frac{\partial (\rho \phi )}{\partial t} + \nabla (\rho v) = 0$$

where $\rho$ is gas density, v indicates gas velocity, and $\phi$ indicates porosity.

Assuming that gas flow rate follows Darcy’s Law,

2 $$v = - \frac{k}{\mu }\nabla p$$

where p indicates pressure, k permeability, and $\mu$ viscosity.

Gas density is dependent on pressure and temperature as follows:

3 $$\frac{\rho }{{\rho_{sc} }} = \frac{{pz_{sc} T_{sc} }}{{p_{sc} zT}}$$

where sc is short for stand condition, and z is compressibility coefficient, function of pressure and temperature.

Integrating Eqs. (2) and (3) into (1),

4 $$\frac{k}{\phi }\nabla \cdot \left( {\frac{p}{\mu Z}\nabla p} \right) = \frac{\partial }{\partial t}\left( {\frac{p}{Z}} \right)$$

The right hand of Eq. (4) could be expanded as follows:

5 $$\frac{\partial }{\partial t}\left( {\frac{p}{Z}} \right) = \frac{p}{Z}\frac{\partial p}{\partial t}\left( {\frac{1}{p} - \frac{1}{Z}\frac{\partial Z}{\partial p}} \right)$$

According to definition of volume compressibility coefficient and combing with Eq. (3),

6 $$c_{g} = \frac{1}{\rho }\left( {\frac{\partial \rho }{\partial p}} \right) = \frac{1}{p} - \frac{1}{Z}\frac{\partial Z}{\partial p}$$

Substituting Eq. (6) into Eq. (5),

7 $$\frac{\partial }{\partial t}\left( {\frac{p}{Z}} \right) = c_{g} \frac{p}{Z}\frac{\partial p}{\partial t}$$

Introducing pseudo-pressure,

8 $$m(p) = 2\int_{{p_{m} }}^{p} {\frac{p}{\mu Z}} {\text{d}}p$$

where $p_{m}$ is reference pressure with value of 0 or 0.1 MPa.

According to Eq. (8),

9 $$\nabla m = \frac{2p}{\mu Z}\nabla p$$

10 $$\frac{\partial m}{\partial t} = \frac{2p}{\mu Z}\frac{\partial p}{\partial t}$$

Substituting Eq. (9) into the left hand of Eq. (4) and Eq. (10) into Eq. (7),

11 $$\frac{k}{\phi }\nabla \cdot \nabla m = c_{g} \mu \frac{\partial m}{\partial t}$$

Then

12 $$\nabla^{2} m = \frac{{c_{g} \mu \phi }}{k}\frac{\partial m}{\partial t}$$

For linearization of Eq. (12), assume that $\mu \phi$ equals approximately to $\overline{\mu \phi }$ which is value under average formation pressure $\bar{p}$.

For steady flow, constant pressure boundary conditions were adopted $\nabla^{2} m = 0$. And in the polar coordinate system, it can be derived by:

$$\frac{{d^{2} m}}{{dr^{2} }} + \frac{1}{r}\frac{dm}{dr} = 0$$

13 $$\begin{aligned} m = m_{e} ,r = r_{e} \hfill \\ m = m_{w} ,r = r_{w} \hfill \\ \end{aligned}$$

where the *r*_e_ is the drainage radius of the elliptical flow, *r*_w_ is the radius of the well. And *m*_e_ is the pseudo-pressure on the boundary of elliptical flow, *m*_w_ is the pseudo-pressure of the well.

Assume that *k* can be described by $k_{{\rm max} }$, $k_{{\rm min} }$ under the polar coordinates as follows:

14 $$k(\varphi ) = k_{{\rm max} } - (k_{{\rm max} } - k_{{\rm min} } )\left| {\sin (\varphi )} \right|$$

where $k_{{\rm max} }$ and $k_{{\rm min} }$ are maximum and minimum sand permeability oriented in *x* and *y* directions, as shown in Fig. 1.

Solving Eq. (13),

15 $$m - m_{w} = \frac{{m_{e} - m_{w} }}{{ln\left( {\frac{{r_{e} }}{{r_{w} }}} \right)}}ln\frac{r}{{r_{w} }}$$

Integrating Eqs. (2), (14), and (15), volumetric flow rate under standard conditions could be obtained:

16 $$Q_{sc} = \frac{{4\pi z_{sc} T_{sc} h\left[ {k_{{\rm max} } + (\frac{\pi }{2} - 1)k_{{\rm min}}} \right]\left( {p_{e}^{2} - p_{w}^{2} } \right)}}{{p_{sc} zT\mu \ln \frac{{r_{e} }}{{r_{w} }}}}$$

### Production rate of a fractured vertical well

In the presence of artificial fracture, the radial flow will be replaced by elliptical flow. With the fracture direction as *x* axis direction, as shown in Fig. 1, Cartesian coordinates, (*x*′, *y*′) can be transformed into elliptical coordinates, using the following relationship:

17 $$\left\{ \begin{array}{l} x = L\cosh \xi \cos \eta \hfill \\ y = L\sinh \xi \sin \eta \hfill \\ \end{array} \right.$$

where $\xi$ and $\eta$ separately represents a family of confocal ellipses and a family of confocal hyperbolas with 2*L* (length of fracture) as focal length (Lou et al. 2013). Assume that the production rate in the elliptical area of the fractured vertical well will follow Darcy’s law:

18 $$dQ_{sc} = \frac{{pz_{sc} T_{sc} }}{{p_{sc} zT}}dA\frac{k}{\mu }\frac{dp}{{d\bar{r}}}$$

Using Eq. (17) dA can be obtained under elliptical coordinate:

19 $$dA = hrd\theta = h\sqrt {x^{2} + y^{2} } d\theta = hL\cosh \xi \cos \eta \sqrt {1 + (\tanh \xi \tan \eta )^{2} } d\theta$$

Applying the relationship between central angle *θ* and eccentric angle $\eta$ of an ellipse:

20 $$\tan \eta = \coth \xi \tan \theta$$

dA can be further derived as a function of $\xi$ and *θ*

21 $$dA = hL\cosh \xi \cos \eta \sqrt {1 + (\tan \theta )^{2} } d\theta = \frac{{hL\cosh \xi \sqrt {1 + (\tan \theta )^{2} } }}{{\sqrt {1 + (\coth \xi \tan \theta )^{2} } }}d\theta$$

Incorporating $\varphi = \theta + \alpha$ into Eq. (14), we can obtain

22 $$k(\theta ) = k_{{\rm max} } - (k_{{\rm max} } - k_{{\rm min} } )\left| {\sin (\theta + \alpha )} \right|$$

Since the elliptical flow is axial symmetrical, assume that the pressure distribution in $\xi$ does not change with $\eta$. Under steady state, the pressure obeys Laplace’s equation in $\xi$ and $\eta$ plane. Therefore the pressure distribution can be described as:

23 $$p = a\xi + b$$

The constants *a* and *b* in the equation are to be determined from boundary conditions

24 $$\left\{ \begin{array}{l} p = p_{w} \, \,at \, \,\xi = 0\,\left( {{\text{the}}\,{\text{fracture}}} \right), \\ p = p_{e} \, \, at \,\, \xi = \xi_{e} \left( {{\text{outer}}\,{\text{boundary}}\,{\text{of}}\,{\text{the}}\,{\text{drainage}}\,{\text{area}}} \right) \\ \end{array} \right.$$

${\kern 1pt} {\kern 1pt} \xi_{e} {\kern 1pt}$ is related to drainage radius $r_{e}$ by:

25 $$\xi_{e} = \ln \frac{{2r_{e} }}{L}$$

Since *p* is only a function of $\xi$, we introduce a modified variable $\bar{r}$ in Darcy’s law, as shown in Eq. (20), where $\bar{r}$ is defined as:

26 $$\bar{r}(\xi ) = \frac{1}{2}L(\cosh \xi + \sinh \xi ) = L \cdot \frac{{e^{\xi } }}{2}$$

Therefore,

27 $$d\bar{r} = \frac{{d\bar{r}}}{d\xi }d\xi = L \cdot \frac{{e^{\xi } }}{2}d\xi$$

Incorporating Eqs. (23), (24), (25), (27) into Eq. (20) will derive:

28 $$dQ_{sc} = \frac{{z_{sc} T_{sc} h}}{{p_{sc} zT}} \cdot \left( {\frac{{p_{e} - p_{w} }}{{\xi_{e} }} \cdot \xi + p_{w} } \right) \cdot \frac{2}{{e^{\xi } }} \cdot \frac{{\cosh \xi \sqrt {1 + (\tan \theta )^{2} } }}{{\sqrt {1 + (\coth \xi \tan \theta )^{2} } }} \cdot \frac{k(\theta )}{\mu }d\theta \cdot \frac{dp}{d\xi }$$

In steady state, the changes of $Q$ with $\xi$ is negligible, thus we can assume $\xi$ as constant when deriving the final expression of $Q_{sc}$:

29 $$\begin{aligned} Q_{sc} = \frac{{z_{sc} T_{sc} h}}{{p_{sc} zT\mu }} \cdot \left(\frac{{p_{e} - p_{w} }}{{\xi_{e} }} \cdot \xi + p_{w} \right) \cdot \frac{2}{{e^{\xi } }} \cdot \left(\frac{{p_{e} - p_{w} }}{{\xi_{e}}}\right) \cdot \cosh \xi \cdot A \hfill \\ where\,A = \int_{0}^{2\pi } {\frac{{\cosh \xi \sqrt {1 + (\tan \theta )^{2} } }}{{\sqrt {1 + (\coth \xi \tan \theta )^{2} } }} \cdot (} k_{1} - (k_{1} - k_{2} )\left| {\sin (\theta + \alpha )} \right|)d\theta \hfill \\ \end{aligned}$$

### Calculation processes

**Fig. 2:**
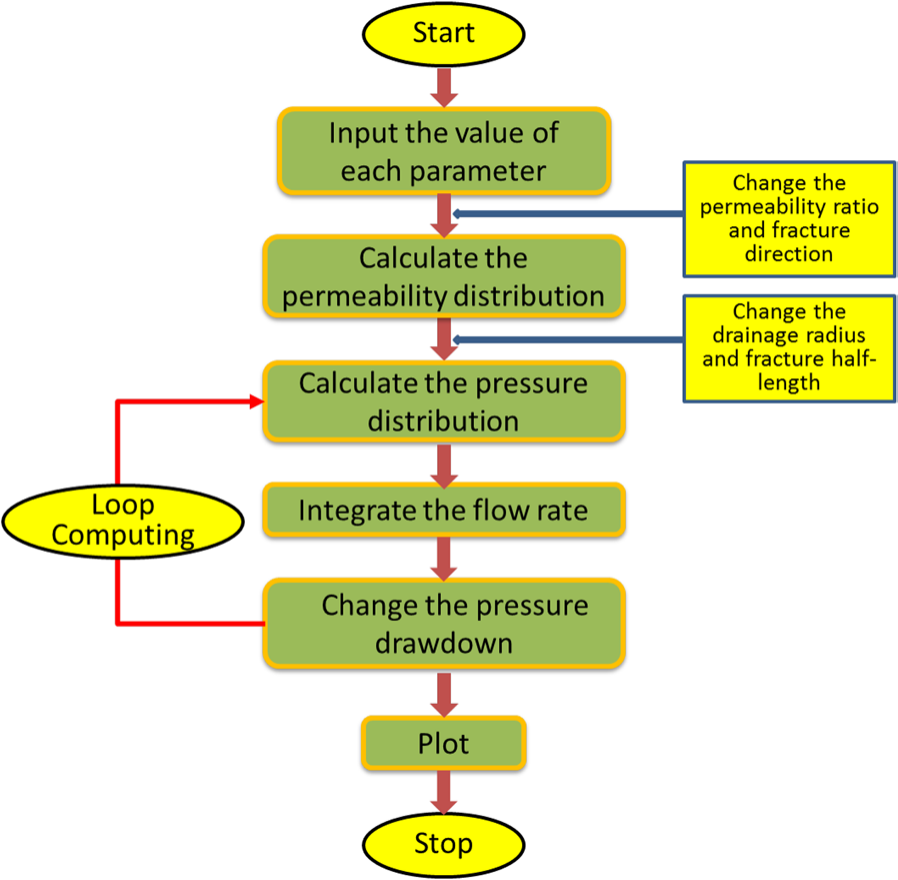
Calculating process for vertical fractured wells

According to the established productivity equations of vertical wells and vertical fractured wells in channel sand tight gas reservoir, the calculation process is carried out using software Matlab. For fractured vertical wells, the calculation process is shown in Fig. 2.

Based on the known parameters, permeability distribution should be first derived by Eq. (22), different formation permeability ratio and fracture direction will directly influence the distribution of permeability. Pressure distribution can be obtained by Eqs. (23) and (24), the values of drainage area and fracture half-length will influence the parameter of Eq. (23), and thus influence the initial pressure distribution. With permeability and pressure distribution being settled, a loop computing of production rate, following Eq. (29), is introduced to obtain the production rate at each pressure drawdown. Finally, the data are output and drawn as diagrams.

## Results and discussion

According to the established productivity equations of vertical well and vertical fractured well, the numerical simulation was carried out. Basic parameters for the numerical simulation are shown in Table 1. Some parameters such as thickness of reservoir, temperature of reservoir, pressure of boundary and bottom hole, maximum and minimum permeability and half-length of fracture were measured or tested in the in-suit and laboratory. Other constants were from the references.

**Table 1:** Basic parameters for numerical simulation of a channel sand reservoir

Parameters	Value (unit)	Parameters	Value (unit)
Reservoir thickness	10 (m)	Pressure at boundary	20 (MPa)
Gas viscosity	0.01 (mPa s)	Bottom hole pressure	12 (MPa)
Reservoir temperature	383 (K)	Drainage radius	1000 (m)
Temperature at std. state	293 (K)	Maximum permeability	5 × 10^−15^ (m^2^)
Pressure at std. state	0.1 (MPa)	Minimum permeability	0.1 × 10^−15^ (m^2^)
Gas Z factor	0.89	Fracture half-length	100 (m)
Gas Z factor std. state	1		

### Comparison between vertical well and fractured vertical well

**Fig. 3:**
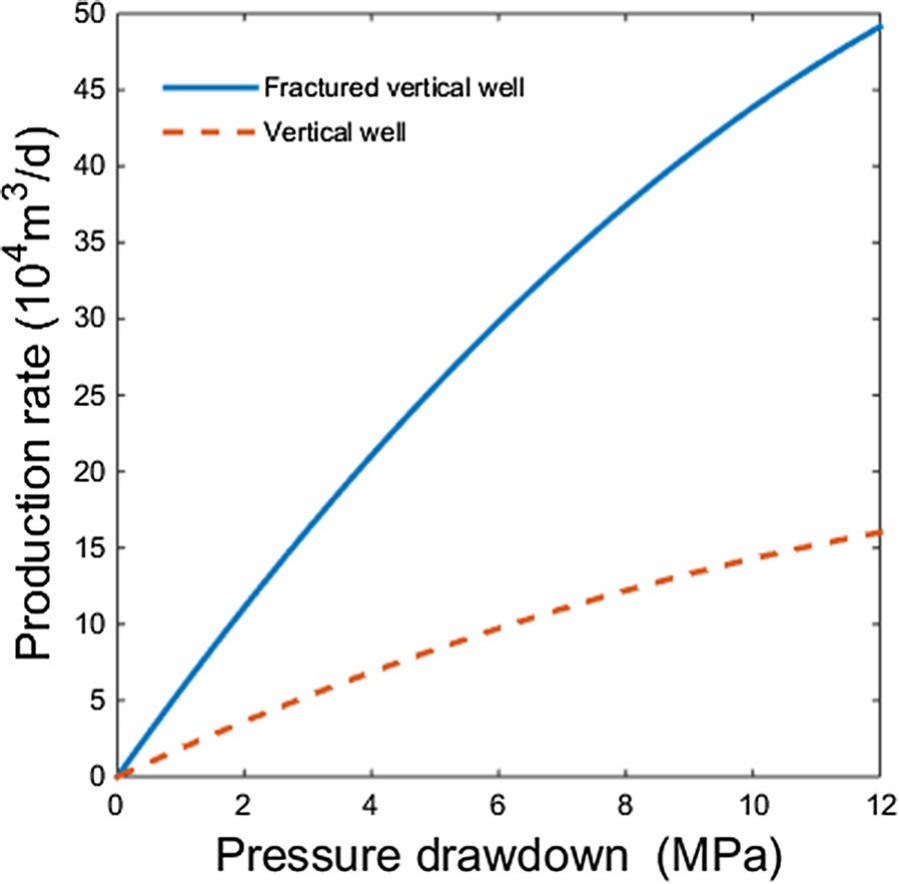
Production rate at different pressure drawdown for vertical well and fractured vertical well

Figure 3 shows the relationship between pressure drawdown and production rate for the two cases discussed in mathematical model. It is obvious that the production rate of fractured vertical well far more exceeds the vertical well without fractures. The difference is much more obvious as pressure drawdown increases.

### Comparison between isotropic and anisotropic formation

**Fig. 4:**
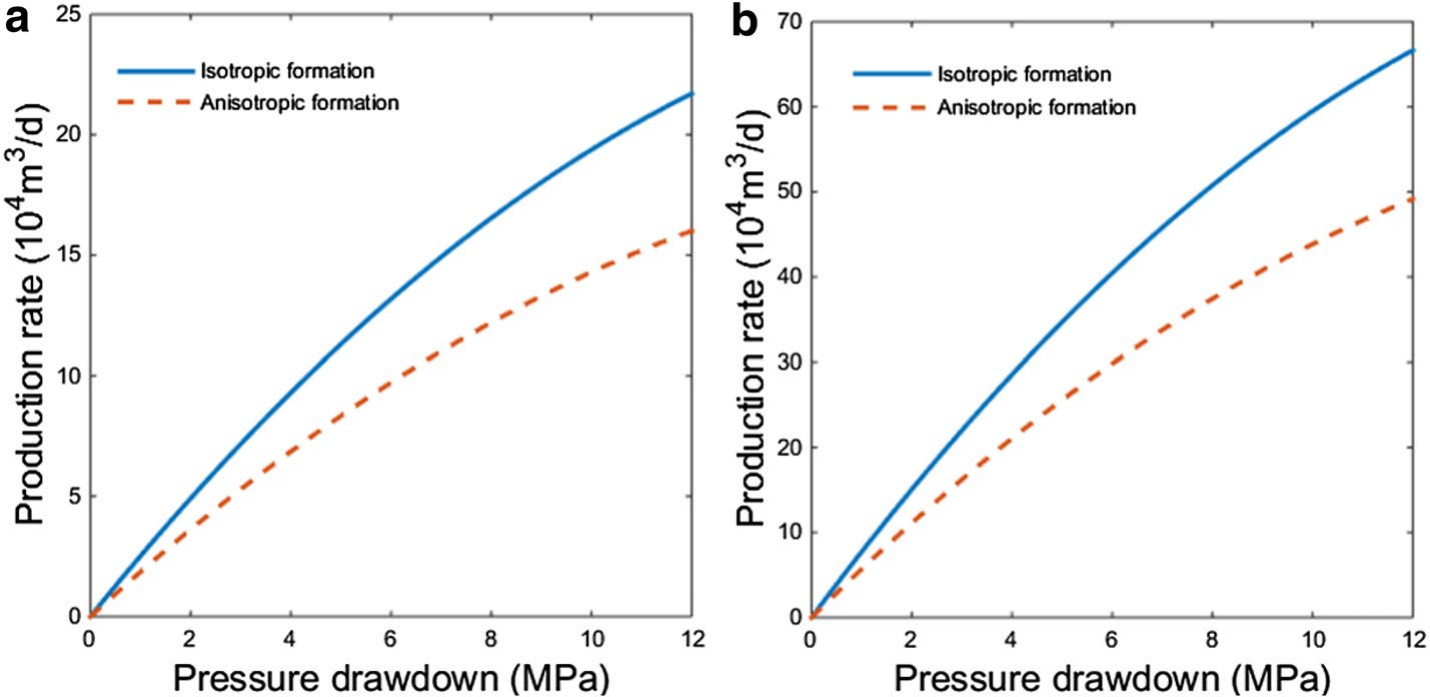
Production rate of isotropic formation and anisotropic formation at different pressure drawdown **a** vertical wells; **b** fractured vertical wells

If taking permeability as a constant, equaling to the average of maximum permeability and minimum permeability, we can gain a comparable production rate and pressure drawdown relationship for an isotropic formation with other parameters setting same as channel sand formation. Figure 4a, b separately show the case of vertical well and fractured vertical well. For both cases, isotropic formation will hold larger production rate. It can be explained similar to the relationship between electric current and conductance. With the total conductivity being settled, the more average the conductivity distribution on each circuit, the larger the current.

### The influence of fracture direction

**Fig. 5:**
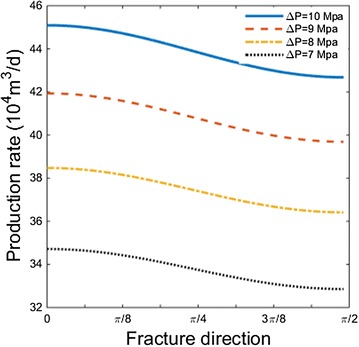
Relationship between fracture direction and production rate for cases with different pressure drawdown

Figure 5 shows the relationship between fracture direction ($\alpha$) and production rate for cases with different pressure drawdown. As fracture direction changes from maximum permeability to minimum permeability, the production rate declines. In an angular range of 0 ~ π/8, we can gain the maximum production rate. For different pressure drawdown, the decline of production rate holds almost the same trend. We explain the phenomenon as follows: in the presence of fracture, the gas flow will change from radial flow to elliptical flow. In this case, the fracture works as a line-type sucking machine for gas, making most gas flow in the same direction as fracture. Thus when the fracture direction follows the maximum permeability direction of the formation, the production rate will be the maximum.

### The influence of drainage radius

**Fig. 6:**
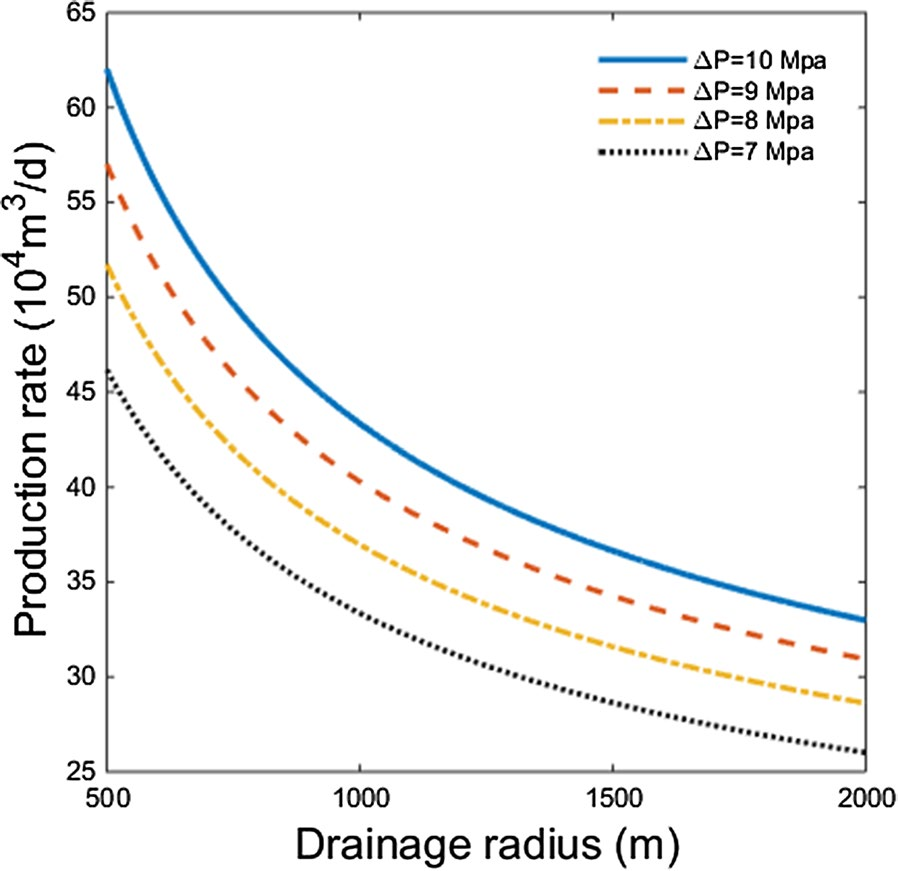
Relationship between drainage radius and production rate for cases with different pressure drawdown

Figure 6 shows the relationship between drainage radius and production rate for cases with different pressure drawdown.

As drainage radius increases, the production rate at first declines rapidly, then declines more smoothly. Besides, the pressure drawdown has no big influence on the decline trend. It is because of the fact that with pressure drawdown settled, the energy for fluid flow is as well steady. As drainage radius increase, the energy gradient via space will decrease, so will reduce the production rate.

### The influence of permeability ratio

**Fig. 7:**
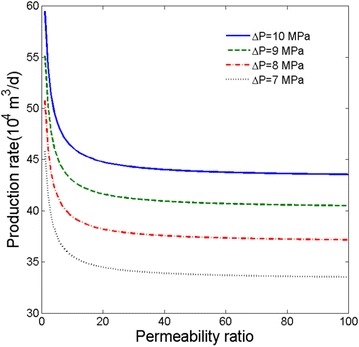
Relationship between permeability ratio ($\frac{k_{\rm max}} {k_{\rm min}}$) and production rate for cases with different pressure drawdown

The permeability ratio is defined as maximum permeability divided by minimum permeability. Figure 7 shows the relationship between permeability ratio and production rate for cases with different pressure drawdown.

As permeability ratio increases (keeping the average permeability is the constant), the production rate will decrease. At first, the production rate declines with permeability ratio rapidly, then the production rate becomes almost constant. In other words, the production rate has a big relationship with maximum and minimum permeability ratio when keeping the average permeability is the constant. As we known, the permeability ratio means the homogeneousness of the tight gas reservoirs. Therefore, the permeability is more homogeneous when the ratio is smaller, whereas it is more heterogeneous. It can be seen that the production rate of gas in the homogeneous reservoir is bigger than in the heterogeneous one. Plus, as pressure drawdown increases, the production rate of gas will also increase.

### The influence of fracture half-length

**Fig. 8:**
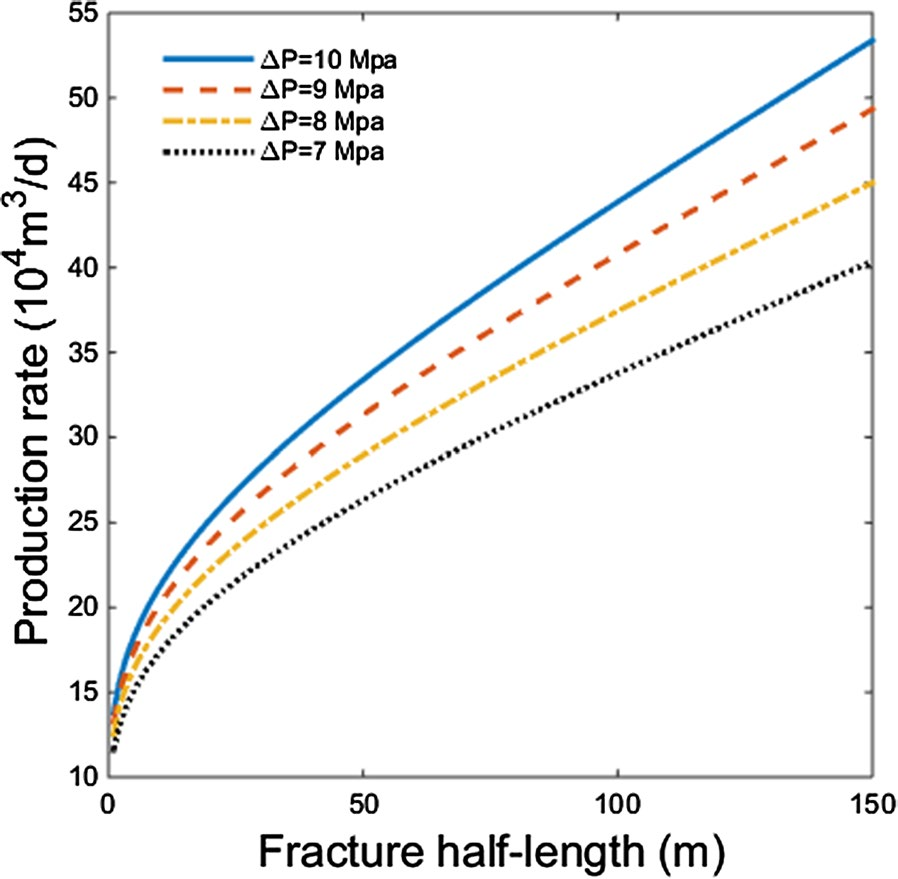
Relationship between fracture half-length and production rate for cases with different pressure drawdown

Figure 8 shows the relationship between fracture half-length and production rate for cases with different pressure drawdown. As fracture becomes longer, the production rate will increase. At first, the increment rate of production declines with fracture length, then the increment becomes almost constant. Pressure drawdown shows little influence on the changing trend.

### In-situ measurement of production rate

According to the analysis above, the factors of influencing production rate were analyzed. In the Table 2, the actual field production data of five fractured wells were listed with respective production conditions which were measured in the Sulige gas reservoir, China. The fractured vertical well was used in all production well. Vertical and horizontal permeability were tested respectively, and the permeability ratio was obtained. In addition, pressure drawdown in every well was measured during production. Therefore, the production rates of each well were calculated with different production conditions. In the Table 2, the average production rates of real field in one month were measured. With comparison of calculated and measured results, the difference rate of anisotropic model is around 12 %, and isotropic model is around 20 %. Hence, the analytical model in tight sandstone gas reservoir with anisotropy permeability can be used to calculate production rate during gas exploitation.

**Table 2:** Comparison of measured data and calculated results

Production well no.	Pressure drawdown/MPa	Tested Max. permeability/m^2^	Permeability ratio	Measured production rate/10^4^ m^3^/day	Calculated production rate/10^4^ m^3^/day			
					Anisotropic	Difference rate/%	Isotropic	Difference rate/%
Shan 319	7.59	0.663 × 10^−15^	9	33.08	37.45	13.2	39.65	19.9
Shan 356	12.01	0.789 × 10^−15^	2	50.39	54.07	7.3	59.63	18.3
Shan 361	10.19	0.054 × 10^−15^	7	10.24	11.65	13.8	13.02	27.1
Su 124	7.99	0.42 × 10^−15^	6	36.95	40.85	10.6	43.36	17.3
Su 192	7.94	0.06 × 10^−15^	8	11.44	12.39	8.3	13.62	19.1

## Conclusions

Based on the above analysis, the following conclusions are put forward:

1. The permeability anisotropy of channel sand formation, mathematical models have been established for both vertical wells and vertical fractured wells. Analytical solutions for production rate considering elliptical flow were derived. For vertical fractured wells, numerical methods were developed to analyze the effect of fracture direction, drainage radius, permeability ratio and fracture half-length on production rate.
2. For both vertical wells and vertical fractured wells, the production rate of anisotropic formation will be less than formation with isotropic permeability.
3. Both the formation properties such as drainage radius and permeability ratio and the fracture properties such as its direction and half-length will influence the production rate considerably. The maximum production rate can be gained when the fracture direction is along or less than π/8 from the maximum permeability.
4. Some actual field measured data were listed and compared with the calculated results. It is shown that the difference rate between measured data and calculated results is acceptable in engineering. Therefore, the analytical model in sandstone gas reservoir can be used in gas recovery.

The combined method of analytical and numerical used by this paper provided a good example of studying this kind of anisotropic tight gas channel sand reservoir.
